# Comparison between intratumoral and intravenously administered oncolytic virus therapy with Newcastle disease virus in a xenograft murine model for pancreatic adenocarcinoma

**DOI:** 10.1016/j.heliyon.2022.e09915

**Published:** 2022-07-09

**Authors:** J. Fréderique de Graaf, Marco Huberts, Daphne Groeneveld, Stefan van Nieuwkoop, Casper H.J. van Eijck, Ron A.M. Fouchier, Bernadette G. van den Hoogen

**Affiliations:** a Viroscience Department, Erasmus Medical Centrum, Rotterdam, the Netherlands; b Department of Surgery, Erasmus Medical Centrum, Rotterdam, the Netherlands

**Keywords:** Newcastle disease virus, Oncolytic viruses, Safety, Treatment efficacy, Intravenous administration, Intratumoral administration, Pancreatic cancer

## Abstract

Pancreatic ductal adenocarcinoma (PDAC) is characterized by a poor clinical prognosis and is usually a metastatic disease. In the last decades, oncolytic viro-immunotherapy has shown a promise as treatment strategy with encouraging results for a variety of tumors. Newcastle Disease Virus (NDV) is an oncolytic virus which selectively infects and damages tumors either by directly killing tumor cells or by promoting an anti-tumor immune response. Several studies have demonstrated that NDV strains with a multi-basic cleavage site (MBCS) in the fusion protein (F) have increased anti-tumor efficacy upon intratumoral injection in murine tumor models. However, intravenous injections, in which the oncolytic virus spreads systemically, could be more beneficial to treat metastasized PDAC in addition to the primary tumor. In this study, we compared the oncolytic efficacy and safety of intratumoral and intravenous injections with NDV containing an MBCS in F (NDV F3aa) in an immune deficient murine xenograft (BxPC3) model for PDAC. In this model, both intratumoral and intravenous injections with NDV F3aa induced anti-tumor efficacy as measured at 10 days after the first injection. Upon intravenous injection virus was detected in some of the tumors, indicating the systemic spread of the virus. Upon both treatments, mice did not display weight loss or abnormalities and treated mice did not secrete virus to the environment. These data demonstrate that intravenous injections of NDV F3aa can be applicable to treat metastasized cancers in immune deficient hosts without inflicting adverse effects.

## 1 Introduction

Pancreatic ductal adenocarcinoma (PDAC) is a cancer with a poor clinical prognosis and high mortality rate. Current approved standard of care of treatment is the resection of the primary tumor if possible, followed by adjuvant chemotherapy with gemcitabine or radiotherapy (Liermann et al., 2022; Neoptolemos et al., 2010). However, the diagnosis of pancreatic cancer is often made at a late stage of the disease when the primary tumor is often already metastasized (Yabar and Winter, 2016). In the case of metastatic spread, the only treatment option left is adjuvant chemotherapy as a palliative therapy to improve quality of life. The use of immunotherapies, such as those with checkpoint inhibitors, has been investigated for treatment of PDAC but has not led to improvement of the current treatment options so far (Hilmi et al., 2018). Oncolytic viro-immunotherapy using oncolytic viruses (OVs) has been explored as a promising new treatment option for of wide variety of cancers (de Graaf et al., 2020; Fukuhara et al., 2016). OVs selectively infect and damage tumors either by directly killing the cells or by promoting an anti-tumor immune response towards them (Harrington et al., 2019). Several oncolytic viruses have been tested in clinical trials with promising results, including Newcastle Disease Virus (NDV). NDV belongs to the family *Paramyxoviridae* and has an avian host range under normal conditions (Alexander, 2008). NDV has shown promise as an oncolytic virus for treatment of a wide range of tumors (Bai et al., 2018; Freeman et al., 2006; Hotte et al., 2007; Keshavarz et al., 2020; Laurie et al., 2006; Mozaffari Nejad et al., 2020; Oseledchyk et al., 2018; Pecora et al., 2002; Ricca et al., 2018). However, studies have also shown that the efficacy of viro-immunotherapies with wild type NDV strains needs improvement (Hotte et al., 2007; Laurie et al., 2006; Pecora et al., 2002). We, and others, aimed to increase the efficacy of treatment with NDV by substitution of the mono-basic cleavage site by a multi-basic cleavage site (MBCS) in the fusion protein (F) of the virus (NDV F3aa) (Buijs et al., 2015; Li et al., 2011; Matuszewska et al., 2019; Silberhumer et al., 2010). This cleavage site alteration results in trypsin independent activation of F and hence an improved replication in multiple cell types, including tumor cells (Alexander, 2008; Buijs et al., 2015). Numerous studies have shown that NDV F3aa had increased oncolytic efficacy compared to wild type NDV when administered intratumorally (IT) in murine models for different cancers (Li et al., 2011; Matuszewska et al., 2019; Silberhumer et al., 2010). The IT route of administration is often used to increase the local viral dose in the tumor and hence obtain improved oncolytic efficacy (de Graaf et al., 2020). In addition, IT injections are hypothesized to be safer than intravenous (IV) injections if the virus is restricted to the tumor. In contrast, IV injections are expected to be more effective in treating metastasized tumors, but the systemic delivery might form a health risk for the patient and its environment. These different opinions about the optimal administration route have resulted in a variety of administration strategies in viro-immunotherapy studies. Two clinical studies have compared safety and efficacy between IV and IT injections using oncolytic Adenovirus (Enadenotucirev) (Garcia-Carbonero et al., 2017) and Parvovirus (ParvOryx) (Geletneky et al., 2017). These studies demonstrated safety and efficacy of the therapy in humans, independent of the route of administration. In addition, these clinical trials showed that application by both administration routes resulted in the presence of viral genomes in the tumor, suggesting that IV injections also result in successful targeting of tumors. This was confirmed in a clinical study applying IV injections with the wild type NDV strain PV701 in patients suffering from advanced chemo refractory cancers, where virus was also detected in the tumor (Pecora et al., 2002).

Our previous study in a PDAC xenograft model demonstrated that IT injections with NDV F3aa induced more tumor regression than injection with wild type NDV, however shedding and viral dissemination to different tissues and the subcutaneous tumor upon treatment was not studied. In that study, tumor growth was assessed during 40 days, after which viral genomes are likely not detected anymore (Buijs et al., 2015). Studies with experimental infections of chickens and cynomolgus macaques with NDV have demonstrated that NDV is only detected in tissues during the first week after inoculation and not at later time points (Buijs et al., 2014a). Here, we aimed to address the question whether IT or IV injections lead to systemic viral distribution. To this end, we compared IT and IV injections, given at days 0, 4 and 8, with NDV F3aa in a PDAC xenograft model for virus distribution and anti-tumor efficacy at day 10 after the first, and 2 days after the last, injection.

## 2 Materials and methods

### 2.1 Cell lines

The PDAC BxPC3 cell line was obtained from American Type Culture Collection (ATTC) and was cultured at 37 °C in Roswell Park Memorial Institute (RPMI) 1640 (Lonza, Switzerland) media supplemented with 100 U ml ^−1^ penicillin, 100 U ml ^−1^ streptomycin, 2 mM L-glutamine (PSG) and 10 % Hyclone Characterized Fetal Bovine Serum (FBS) (Thermo Fischer Scientific, The Netherlands). BSR-T7 (kind gift of K. Conzelmann) were cultured in Dulbecco’s Modified Eagle’s Medium (DMEM, Lonza, The Netherlands) supplemented with PSG and 10 % FBS at 37 °C. Vero cells, obtained from ATCC, were cultured in Iscove Modified Dulbecco Media (IMDM) (Lonza, Switzerland) with the same supplements. Periodically, cells were tested and confirmed to be *mycoplasma* free.

### 2.2 Virus preparation

The plasmid containing the full-length cDNA of lentogenic NDV strain La Sota (pNDV F0) and expression plasmids for the NP, P and L proteins were kindly provided by Prof. B. Peeters from the Central Veterinary Institute of Wageningen, The Netherlands (Peeters et al., 1999). To create the NDV cDNA clone of mesogenic NDV F3aa, the amino acid sequence of the protease cleavage site was changed from ^112^GRQGR↓L^117^ (lentogenic) to ^112^RRQRR↓F^117^ using site-directed mutagenesis as described earlier (Buijs et al., 2015). Recombinant NDVs were rescued using an adapted method from the one described previously (Peeters et al., 1999). Briefly, BSR-T7 cells were transfected with 5 μg full length pNDV, 2.5 μg pCIneo-NP, 1.25 μg pCIneo-P and 1.25 μg pCIneo-L using 8nM calcium phosphate. For NDV F0, three days later, 200 μL BSR-T7 supernatant was injected into the allantoic cavity of 10-day-old specified pathogen free (SPF) embryonated chicken eggs. After incubation in a humidified egg incubator at 37 °C for three days, allantoic fluid was harvested and stored at -80 °C. For F3aa 200 μL BSR-T7 supernatant was used to inoculate Vero cells. Five days later cells and supernatant were harvested and stored at −80 °C. The titer of the virus stock were determined by end-point titration in Vero cells and calculated using the method of Reed & Muench and expressed as TCID_50_ ml ^−1^ (Reed and Muench, 1938).

Virus stocks were generated after the second passage in Vero cells using a multiplicity of infection (MOI) of 0.01, based on the titer of the virus stocks generated during passage 1 and the number of cells plated out in the flasks. Five days after inoculation the virus was harvested and subsequently titrated by end-point titration in Vero cells. In case of NDV F0, 2 μg ml ^−1^ TPCK-treated Trypsin (T1426, Sigma-Aldrich, The Netherlands) was added to the media during infection. Virus batches were stored in 25 % sucrose (w/w) at -80 °C. Virus stocks for *in vivo* studies were concentrated by centrifugation over an Amicon® Ultra – 100 kDa NMWCO (Merck Millipore, UFC9100, Germany) and purified by filtration over a low protein binding filter membrane of 0.45 μM (Merck Millipore, LHV033RS). Mock treatment consisted of supernatant from non-inoculated cells that was purified and concentrated as described for the virus batches. Stocks were store at -80 °C.

### 2.3 Replication curves

One million cells were seeded in 6-well plates (Corning, The Netherlands). The next day, cells were inoculated at an MOI of 0.05 and one hour after inoculation cells were washed three times with phosphate buffered saline (PBS) after which media without FBS or trypsin was added. At indicated time points, 100 μl sample was collected and stored in 25 % sucrose (w/w) at -80 °C. Subsequently, samples were titrated by end point dilution assay in quadruplicate in Vero cells.

### 2.4 Cytotoxicity assay

Quadruplicates of 2 × 10^4^ cells per well in 96-well plates (Greiner, The Netherlands) were either mock inoculated or inoculated with at the indicated MOI. One hour after inoculation, cells were washed once with PBS and fresh media without FBS or trypsin was added. Five days after inoculation, a lactate dehydrogenase assay (LDH, Cytotox 96 Non-Radioactive Cytotoxicity Assay, Promega, The Netherlands) was used to determine cell viability following the manufacturer’s instructions as described before (Buijs et al., 2014b).

### 2.5 Animals and experimental design

In total 32 eight-weeks old, athymic male mice (strain NMRI-*Foxn1*^*nu*^, Charles River, Sulzfeld, Germany) were used. The mice, with an average weight of 30 g, were acclimated to the housing conditions for 7 days, were fed chow and water ad libitum and were housed under specific pathogen-free conditions. Group sizes were determined as suggested by Charan and Kantharia (2013). In brief, a 2-sided t-test was used with a power of 80 %, a significance of 0.05 and expected difference between experimental groups of 25 with a standard deviation of 15, leading to the use of 8 animals per group. After acclimatization, groups of 8 mice, randomly divided over 4 groups based on weight, were inoculated subcutaneously with 5 × 10^6^ human PDAC BxPC3 cells, suspended in culture medium without FCS, while under isoflurane anesthetics. Five weeks later, mice with tumor sizes >15 mm^3^ were selected in each group, resulting in the following treatment groups: (1) 7 animals IT injection with mock; (2) 6 animals IT injection with NDV; (3) 6 animals IV injection with mock and (4) 6 animals IV injection with NDV. IT injections were given under isoflurane anesthetics and intravenous injections were given without anesthetics under a heating lamp. Mice were treated three times every four days with either mock or 1 × 10^5^ TCID^50^ virus by intravenous injection via the tail vein (100 μl) or intratumorally (50 μl). The inoculum was titrated in Vero cells to confirm the injected dosage. Weight was measured until the mice were euthanized. Tumor growth was measured every two days by using a digital calliper. Volume was calculated by the following formula: width^2^ × length/2, as described before (Buijs et al., 2015). Swabs from the urinal duct and approximately one gram of fresh feces (fecal sample) from the bottom of the cage were collected every two days, and a throat swab was taken during necropsy. All samples were collected in 1 ml virus transport media consisting of Hanks balanced salt solution containing 0.5 % lactalbumin, 10 % glycerol, 200 U ml^−1^ penicillin, 200 μg ml ^−1^ streptomycin, 100 U ml^−1^ polymyxin B sulfate, 250 μg ml^−1^ gentamicin, and 50 U ml^−1^ nystatin (ICN, The Netherlands).

### 2.6 Ethics statement

All experiments involving animals were conducted strictly according to the European guidelines (EU directive on animal testing 86/609/EEC) and Dutch legislation (Experiments on Animals Act, 1997). The animal study was reviewed and approved by an independent animal experimentation Dutch ethical review committee (DEC consult number: AVD101002017867).

### 2.7 Collection, processing and storage of tissue and environmental samples

Swabs and feces were collected in virus transport media (500 μl) (Munster et al., 2009). Blood samples were collected in blood collection tubes (Minicollect, Greiner Bio-one, 450533, The Netherlands), centrifuged for 10 min at 250 x *g* and the serum was stored at -20 °C. All organs collected during necropsy were snap frozen and stored at -80 °C. Organs and tumor tissues were supplemented with Dulbecco’s Modified Eagle’s Medium (DMEM, Lonza, The Netherlands) and PSG and subsequently homogenized using a FastPrep 24 tissue homogenizer (MP Biomedicals, The Netherlands). Homogenized samples were centrifuged for 10 min at 2000 x *g* and supernatant was stored at -80 °C or 200 μl was used for Ribonucleic acid (RNA) isolation.

### 2.8 RNA isolation and quantitative real-time polymerase chain reaction (qRT-PCR)

RNA was extracted from the urine, fecal and throat samples by adding 60 μl sample to 90 μl Magnapure 96 external lysis buffer (6374913001, Roche Diagnostics, The Netherlands) as described before (Richard et al., 2020). Subsequently, the lysed sample was added to 60 μl Agencourt AMPure XP magnetic beads (A63880, Beckman Coulter, The Netherlands) and incubated 15 min at room temperature. Magnetic beads were washed three times with 70 % ethanol using the DynaMag-96 magnet (12027, Invitrogen, The Netherlands) and subsequently air-dried. RNA was eluted by 6 min of incubation in bidest H_2_O. NDV-specific quantitative reverse transcription-PCR was performed using 5 μl RNA in an ABI PRISM 7000 Sequence Detection System using TaqMan Fast Virus 1-Step Master Mix (both from Thermo Fischer) in a total volume of 30 μl. The NDV-specific primers used were described by Wise et al. (2004). The reverse transcriptase step was 5 min at 50 °C, followed by 95 °C for 20 s. Cycling consisted of 40 cycles of 3 s denaturation at 95 °C, 5 s annealing at 54 °C and 31 s extension at 60 °C.

### 2.9 NDV serology

Sera were tested for the presence of NDV specific antibodies by hemagglutination inhibition assay (HI) using turkey erythrocytes as described before (Buijs et al., 2014a). Chicken polyclonal NDV antibody (ab34402, Abcam, UK) was used as positive control.

### 2.10 Histopathology and immunohistochemistry

Formalin-fixed, paraffin-embedded, 3-μm-thick sections of the same tissues examined histopathological and were stained using an immunoperoxidase method as described before (Buijs et al., 2014a). Tissue sections were mounted on coated slides, deparaffinized and rehydrated. Sections were incubated in 3 % H_2_O_2_ in PBS for 10 min at RT to block endogenous peroxidase. Subsequently, antigen was retrieved by incubation of the section in Tris–EDTA buffer (pH 9) for 15 min, washed with PBS containing 0.05% Tween 20 and subsequently incubated in PBS with 0.1 % Bovine Serum Albumin (BSA) for 10 min at RT. Next, sections were incubated in PBS with 0.1 % BSA with a monoclonal mouse antibody IgG2a to NDV (dilution 1:100, MAb 6H12, specific to ribonucleoprotein; La Sota strain, Hytest Ltd, Turku, Finland) or with a negative control isotype mouse monoclonal antibody (dilution 1:100, MAb 003, R&D System, Minneapolis, USA) for 1 h at RT. After washing, sections were incubated with goat anti-mouse antibody (dilution 1:400, Southern Biotech, Birmingham, AL, USA) labeled with horseradish peroxidase (HRP) for 1 h at RT. In the next step, the sections were incubated in 3-amino-9-ethylcarbazole (Sigma Chemical Co., St. Louis, USA) in *N*,*N*-dimethylformamide (Sigma Chemical Co.) solution for 10 min at RT, resulting in a red precipitate, which represents HRP activity. The sections were counterstained with hematoxylin. Brain tissue sections from a cormorant (*Phalacrocorax auritus*) known to be infected with NDV were used as a positive control and tissue sections of a mock inoculated mice were used as a negative control. Slides for histopathology were stained with hematoxylin and eosin (H&E) and examined by light microscopy.

## 3 Results

### 3.1 NDV induced cell death of human pancreatic adenocarcinoma (PDAC) cells *in vitro*

**Figure 1:**
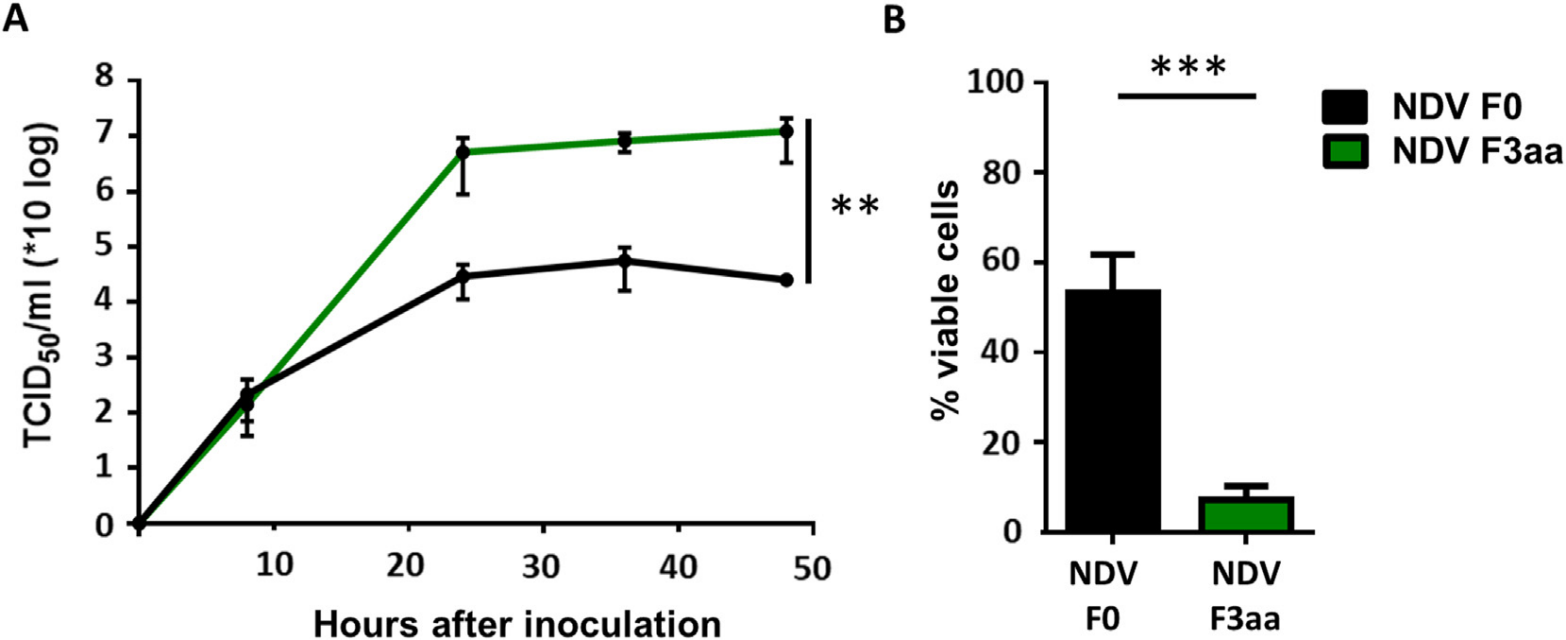
*In vitro* evaluation of BxPC3 cells for susceptibility to NDV infection. **(**A) Replication kinetics of NDV F0 and NDV F3aa in human pancreatic tumor cells BxPC3. Cells were inoculated at an MOI. of 0.05 in triplo. At the indicated time points samples were taken and titrated in Vero cells. The experiment was conducted two times in triplo. Means and standard deviations of triplicates of a representative experiment are plotted. Data was compared between groups using a linear regression analysis (B) Virus induced cell death of BxPC3 cells upon inoculation with NDV F3aa and NDV F0. BxPC3 cells were inoculated at an MOI of 1 in triplo with indicated viruses. Results are represented as percentage viable cells compared to mock, which were considered as 100% viable. The experiment was conducted two times. Means and standard deviations of triplicates of a representative experiment are plotted. Data was compared between groups using an unpaired T test. P values below 0.05 were considered statistically significant and are represented by a ∗. P values below 0.01 or 0.001 are represented by ∗∗ or ∗∗∗.

Human PDAC BxPC3 cells, to be used in the subcutaneous xenograft model, were first assessed for susceptibility to infection with NDV F3aa. Infection with recombinant NDV F0 was taken along to compare the cell killing to that induced by infection with NDV F3aa and for comparison with results of our previous experiments (Buijs et al., 2015). Upon inoculation of the BxPC3 cells, NDV F3aa replicated significantly more efficient than NDV F0 (Figure 1A). In addition, virus induced cell death was significantly higher upon inoculation with NDV F3aa than upon inoculation with NDV F0 (Figure 1B). These data demonstrated that NDV F3aa replicated to high titers in BxPC3 cells and, at 5 days after inoculation, this replication resulted in cell death of more than 80% of the cells which was in line with our previous *in vitro* studies (Buijs et al., 2015).

### 3.2 Anti-tumor efficacy induced by IT and IV injection with NDV F3aa in immune deficient mice subcutaneously inoculated with BxPC3 cells

The anti-tumor efficacy induced by IT or IV injections with NDV F3aa was evaluated in athymic mice subcutaneously inoculated with human PDAC BxPC3 cells. The animals were treated at day 0, 4, and 8 and were euthanized at day 10 after the first injection. This is two days after the final injection, and ten days after the first injection, a time point at which viral genomes could potentially still be detected in the tissues of the treated animals.

**Figure 2:**
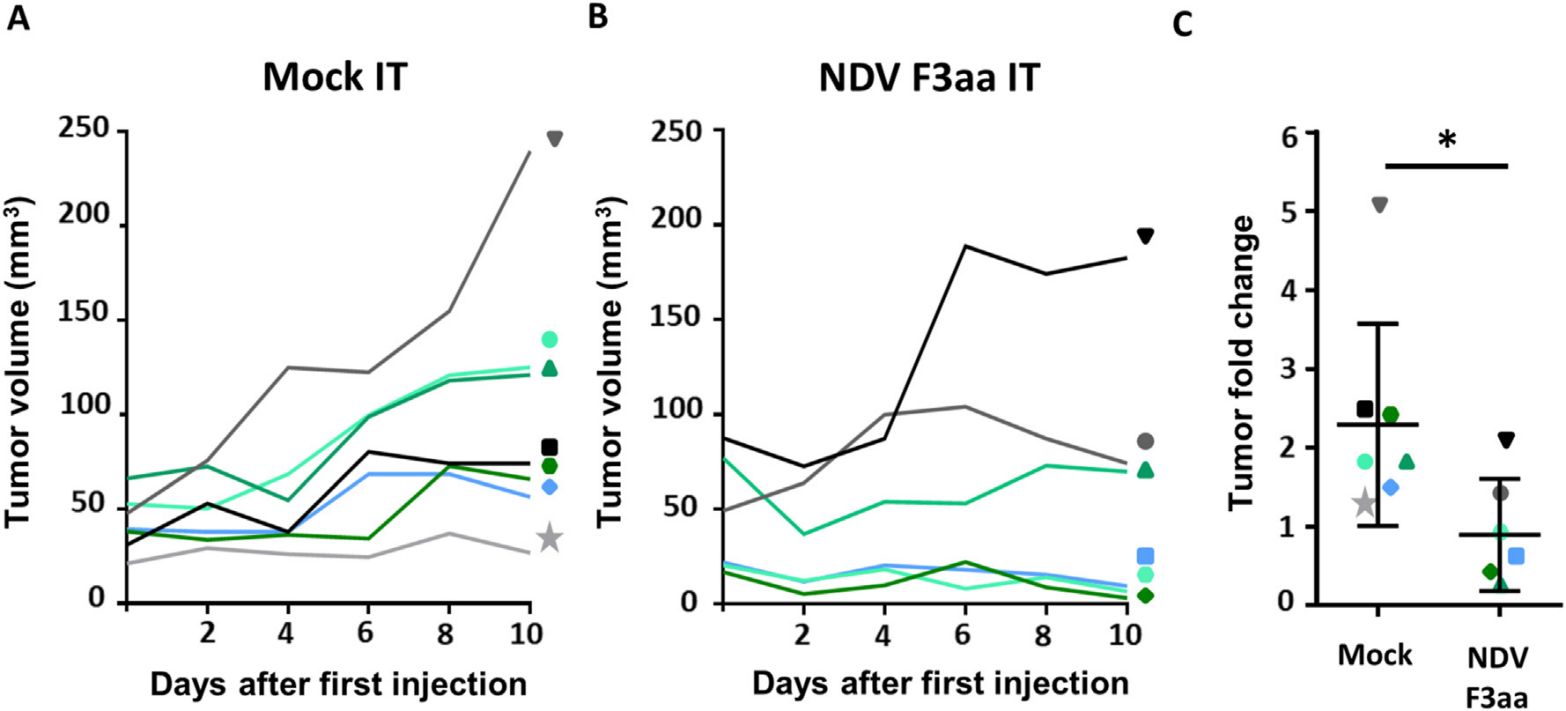
Tumor growth upon intratumoral injection with NDV F3aa in immune deficient tumor bearing mice. **(**A–B): Tumor volumes per individual mice during 10 days after three intratumoral (IT) injections with (A) mock and (B) NDV F3aa (C) Mean fold change in tumor volume and standard deviation between day 0 and 10 per group of treated animals. Each colour and symbol correspond with the individual mice within the mock group or within the NDV F3aa group. Data was compared between groups using a paired T test. P values below 0.05 were considered statistically significant and are represented by an ∗.

At day 10 after the first IT injections, and two days after the last injection, with mock, all mice had increased tumor volumes compared to day 0. In contrast, four out of six mice that had tumors IT injected with NDV F3aa showed decreased tumor volumes at day 10 (Figure 2A-B). On average, the tumors in all seven mice IT injected with mock had a significant higher fold change in tumor volume than the tumors in the six mice IT injected with NDV F3aa (Figure 2C).

**Figure 3:**
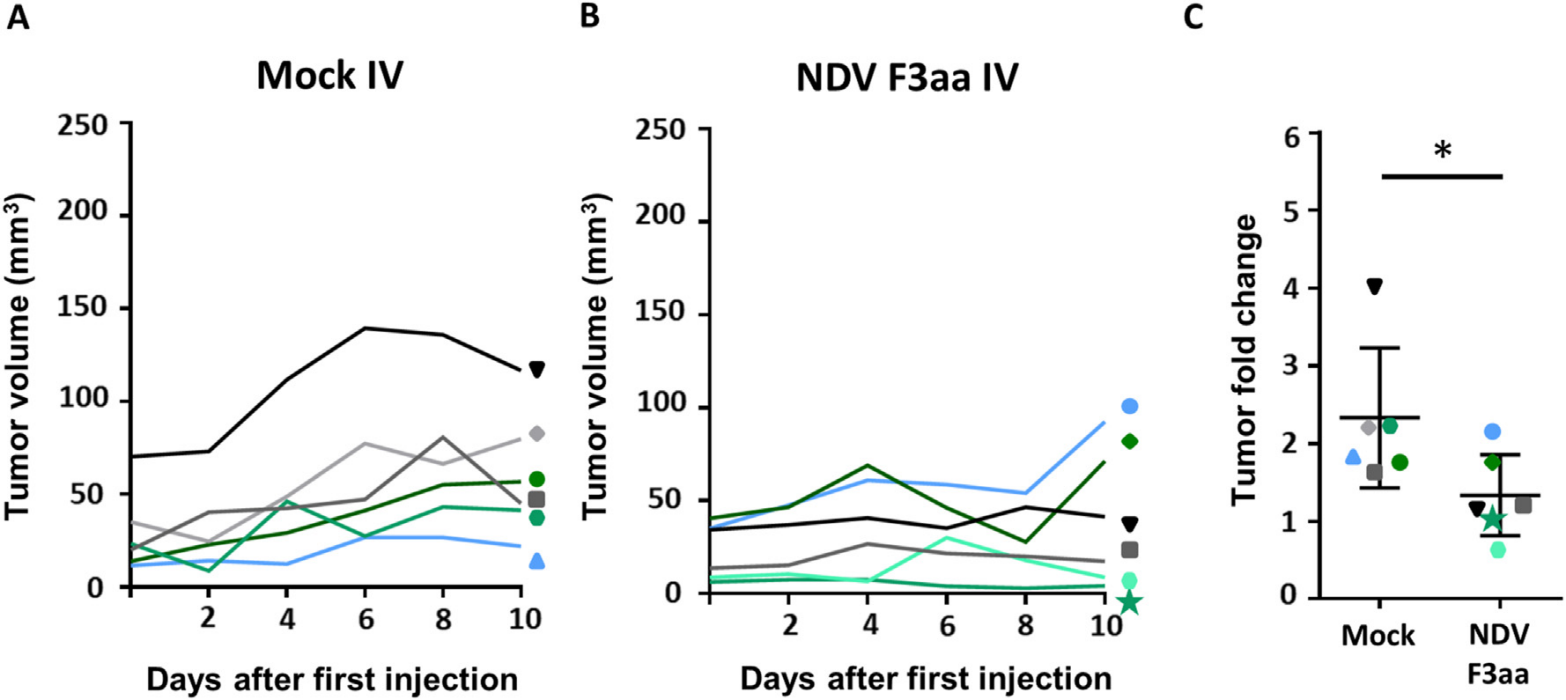
Tumor growth upon intravenous injection with NDV F3aa in immune deficient tumor bearing mice. (A–B): Tumor volumes per individual mice during 10 days after three intravenous (IV) injections with (A) mock and (B) NDV F3aa (C) Mean fold change and standard deviation in tumor volume between day 0 and day 10 per group of treated animals. Each colour and symbol correspond with the individual mice within the mock group or within the NDV F3aa group. Data was compared between groups using a paired T test. P values below 0.05 were considered statistically significant and are represented by an ∗.

At day 10 after the first IV injection with mock, and two days after the last injection, all mice had increased tumors volumes compared to day 0, while in only two out of six animals IV injected with NDV F3aa the tumors increased in volume. In the other four animals IV injected with NDV F3aa, the tumor volumes slightly decreased or remained similar in size (Figure 3A-B). On average, the fold change in tumor volume at 10 days after treatment was significant smaller in animals IV injected with NDV F3aa than in animals IV injected with mock (Figure 3C). These data show that both IV and IT injection with NDV F3aa have anti-tumor efficacy in immune deficient mice subcutaneously inoculated with PDAC cells, at least at ten days after the first injection.

### 3.3 Virus dissemination upon IT or IV treatment

**Figure 4:**
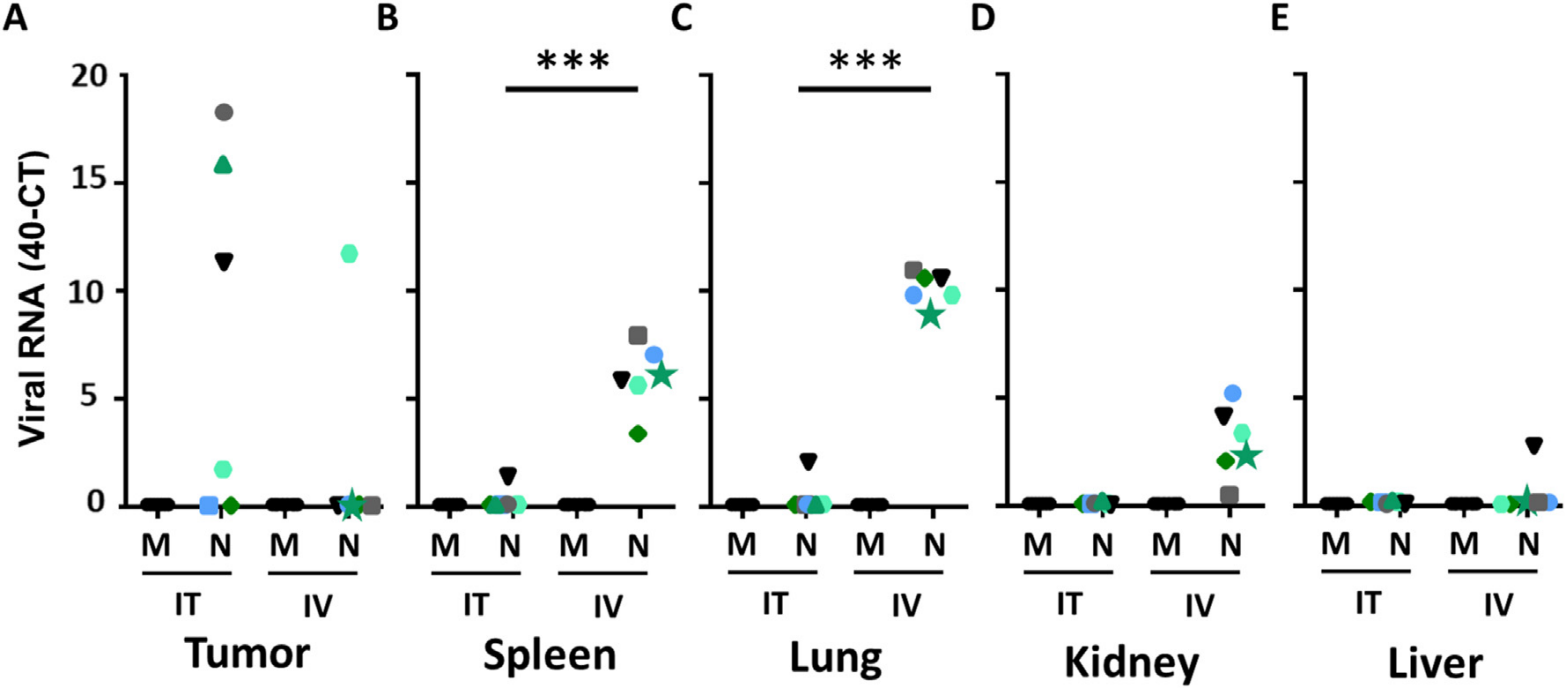
Dissemination of viral RNA in immune deficient tumor bearing mice upon intratumoral or intravenous injection with NDV F3aa. Detection of viral genomes in (A) tumor, (B) Spleen, (C) Lung, (D) Liver and (E) Kidney collected from mice at day 10, after three intratumoral (IT) or intravenous (IV) injections with mock (M) or NDV F3aa (N). Values are shown in ΔCT (40-CT): higher values for ΔCT indicate the presence of higher levels of viral genomes in the samples. Each colour and symbol correspond with the individual mice represented in Figures 2 and 3. Data was compared between groups using an unpaired T test. P values below 0.05 were considered statistically significant and are represented by an ∗. P values below 0.01, 0.001 or 0.0001 are represented by ∗∗, ∗∗∗ or ∗∗∗∗.

**Fig. S1:**
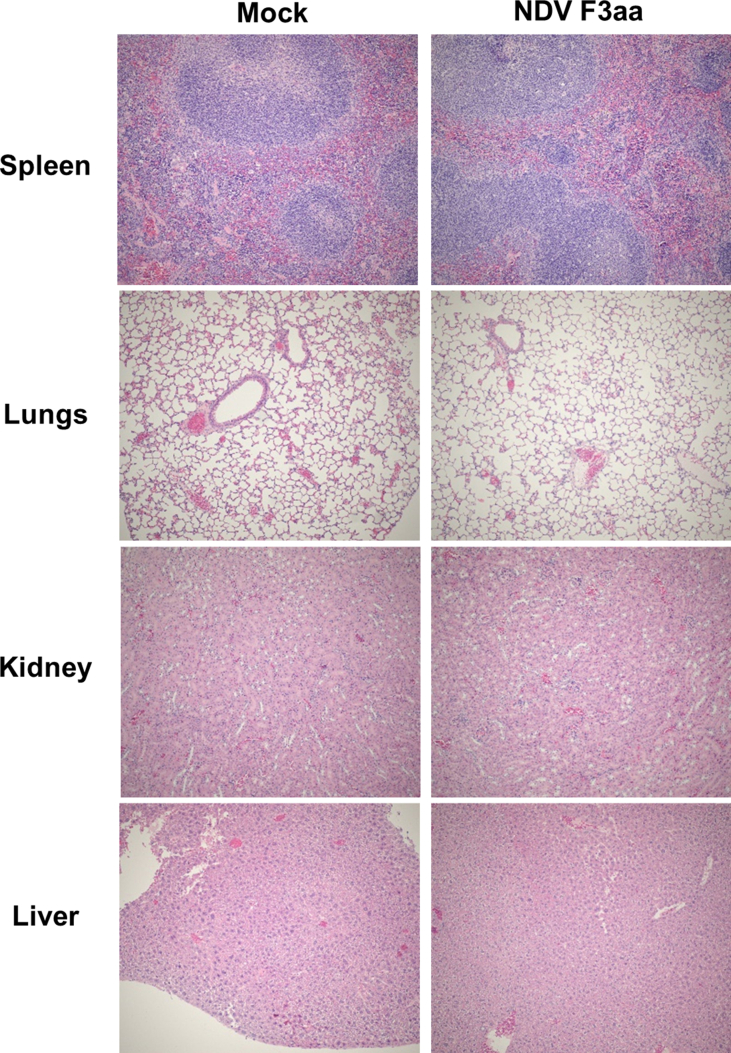
**Histopathology of spleen, lung, kidney and liver of immune deficient tumor bearing mice upon intravenous injection with NDV F3aa.** (A) Representative images of an H&E staining of tissues of immune deficient mice injected with mock or NDV F3aa (original magnification 10x).

To compare viral dissemination between IV and IT injection with NDV F3aa in immune deficient tumor bearing mice, the presence of viral RNA in tumors, lungs, spleen, liver and kidneys at day 10 after the start of treatment, and two days after the last injection, was determined. Upon three IT injections with NDV F3aa, viral RNA was detected in four out of six injected tumors, while upon three IV injection viral genomes were only detected in the tumor of one treated animal. Upon IT injections with NDV F3aa, viral genomes were not detected in any other organ than the tumor in five out of six mice. In the sixth IT injected animal, viral genomes were also detected in the spleen and lung, but not in the liver and kidney. In contrast, upon three IV injections with NDV F3aa, significant higher levels of viral RNA were detected in the spleen and lung of all animals compared to those in IT injected animals and viral RNA was detected in the kidney of five IV injected animals and the liver of one IV injected animal (Figure 4). Subsequently, immunohistochemistry was used to detect viral protein in those organs positive for viral RNA as indication for viral replication. Using brain tissue sections from a cormorant (*Phalacrocorax auritus*) known to be infected with NDV as a positive control and tissue sections of a mock inoculated mice as a negative control, viral protein expression was not observed in any of the RNA-positive organs nor were any pathological abnormalities observed (Supplementary figure 1).

**Figure 5:**
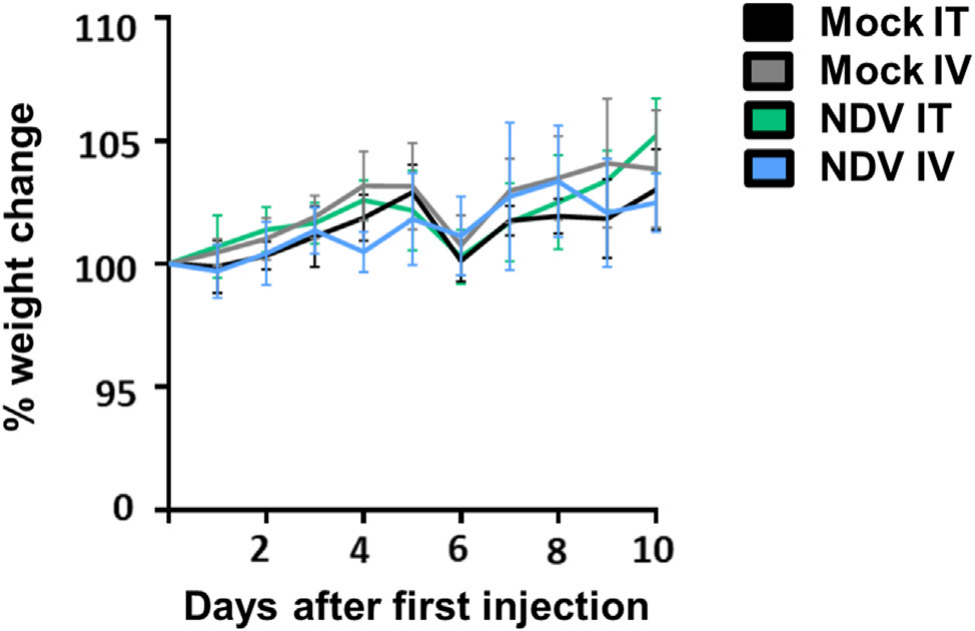
**Weight changes in immune deficient tumor bearing mice upon intratumoral or intravenous injection with NDV F3aa.** Average weight per group and standard deviation. Weight of each animal was measured and depicted as normalized average weight per group. Black line: average of animals IT injected with mock, grey line: average of animals IV injected with mock, green line: average of animals IT injected with NDV F3aa, blue line: average of animals IV injected with NDV F3aa. Data was compared between groups using a linear regression analysis. P values below 0.05 were considered statistically significant. No significant differences were observed.

The average weight of the virus treated mice, either IT or IV injected, did not differ significantly from the average weight of the mock treated mice and none of the mice showed weight loss during the 10 days of treatment (Figure 5). In addition, viral RNA was not detected in urine, throat and feces samples, collected from the injected animals, indicating that both IT and IV virus injected mice did not shed detectable amounts of virus in the environment. None of the IV or IT treated mice showed seroconversion. These data suggests that IV and IT injections with NDV F3aa did not induce any adverse effects in these immune deficient animals even though viral RNA was detected in several organs in IV virus injected animals.

In conclusion, IV and IT injection with NDV F3aa in this xenograft murine model for PDAC resulted in reduced tumor growth during ten days after start of the treatment and IV injection did result in targeting tumors systemically in one of the animals. In addition, both IV and IT injections with NDV F3aa in this model did not lead to adverse effects in the mice and neither to shedding of virus to the environment.

## 4 Discussion

Oncolytic viro-immunotherapy is a form of cancer immunotherapy for which research has shown promising results in preclinical and clinical studies for treatment of a variety of tumors (Bommareddy et al., 2018; Lohmueller and Finn, 2017). Previously, we have shown that IT injections with NDV F3aa had higher oncolytic efficacy than injections with NDV without an MBCS in this same xenograft model, as measured at 40 days after start of the treatment (Buijs et al., 2015).

Numerous preclinical and clinical studies for oncolytic viro-immunotherapy have been reported, but the routes of administration, such as IV and IT injections, vary greatly and are sparsely compared side by side (de Graaf et al., 2020). Patients with PDAC are often diagnosed at a time point that the disease is already metastatic. The systemic spread of tumor cells would argue for IV administration of viro-immunotherapy, in addition to the fact that application of IT injection of pancreas tumors would be challenging. However, upon IV administration the oncolytic virus might not reach the tumor, which would reduce anti-tumor efficacy. In addition, IV administration of NDV F3aa could induce adverse effects upon replication in healthy tissues and the systemic spread of the viruses might cause a problem for the environmental safety. Some reports have suggested that locoregional viral administration, via hepatic arterial infusion or via a split-spleen reservoir, offers a more tumor specific treatment modality, without the need for large systemic viral loads thereby minimizing systemic toxicity. However the full potential of locoregional application of virotherapy has yet to be investigated (Apostolidis et al., 2007; Carpenter et al., 2010; Kooby et al., 1999). So far, IV and IT injections with NDV F3aa have not been compared side by side to evaluate the differences between anti-tumor efficacy and safety, such as viral dissemination and virus shedding. Here, we made this comparison with NDV F3aa in an immune deficient subcutaneous xenograft model for PDAC, during 10 days of treatment. For this study, we used the NDV susceptible human PDAC BxPC3 cell line to induce tumor growth in an immune deficient murine model (Buijs et al., 2015).

In the mice IT injected with NDV F3aa, reduced tumor growth was observed compared to mock treated mice, at 10 days after the first, and 2 days after the last, injection. As IT injection might have caused an initial local inflammatory response, the reduction in tumor volumes might have been underestimated. These observations were in line with other studies in which NDV F3aa was IT injected in xenograft murine models for different tumors, such as head and neck cancer, ovarian cancer and mesothelioma models (Buijs et al., 2015; Li et al., 2011; Liu et al., 2020; Matuszewska et al., 2019; Silberhumer et al., 2010). In these studies, tumor growth was followed for more than ten days allowing to address anti-tumor immune responses, but virus dissemination and shedding was not addressed.

Similar as upon IT treatment, IV injection with NDV F3aa in our xenograft model reduced the growth of subcutaneous tumors compared to treatment with mock during the 10 days of treatment, indicating that systemically applied virus is effectively targeting tumors in the flank of the animals. Although these results were obtained in immune deficient mice, similar results have been reported for an immune competent glioma murine model in which IV injections improved survival of mice bearing glioma tumors (Koks et al., 2015).

In this study, a dose of 1 × 10^5^ TCID_50_ NDV generated in Vero cells was used. A dose of 1 × 10^5^ TCID^50^ has been shown to be effective upon application in a syngeneic model for pancreatic adenocarcinoma (Schwaiger et al., 2017). It has previously been shown that egg-produced NDV is partially neutralized by the mammalian complement system (Biswas et al., 2012; Rangaswamy et al., 2016), hence the effective dose used in the present study may be higher than reported by Schwaiger et al. Future studies have to show whether the efficacy indeed increase with the use of mammalian-derived NDV. Upon IT injections, viral genomes were only detected in the tumors and not in other tissues, while IV treatment resulted in the presence of viral RNA in several organs, but to a lesser extent in the tumor tissues. That IV injection leads to viral expression spleens, in contrast to IT injection, might be beneficial for activation of the anti-tumor immune response, but this has to be confirmed in an immune competent tumor model. These data show that, in contrast to IT injection, IV treatment results in systemic delivery of the virus and hence potential effective targeting of metastatic tumor sites**.** These data suggest that detection of virus in the tumors is not indicative for oncolytic efficacy. The mechanism of action for viro-immunotherapy relies not only on direct oncolysis but also on activation of the innate and adaptive immune system (de Graaf et al., 2020). The NDV treated animals did not develop an anti-viral antibody response as the duration of the experiment was too short for the animals to develop such a response. In addition, the used athymic mice lack T cells and as a result have B cells with a reduced function. However, it has been demonstrated that NK cells can still function in these immune deficient mice (Dorshkind et al., 1985). Virus induced activation of the NK cells could be the mechanism resulting in the observed reduced tumor growth in our model, but more detailed research is necessary to prove this mechanism of action in immune deficient mice.

IV injections with NDV F3aa significantly decreased tumor growth compared to mock treated mice, however tumor growth was only evaluated until day ten post inoculation. Further studies should be conducted to determine the effect of IV injections with NDV F3aa on the health of mice and tumor regression at later time points in both immune deficient and immune competent murine models for PDAC.

Different studies in immune competent murine tumor models have shown that IV injections with NDV strain La Sota (a wildtype nonvirulent strain) or strain Italien (a virulent strain) resulted in virus expression in subcutaneous breast cancer tumors or in metastatic sites in the liver (Liu et al., 2020; Wei et al., 2015). The detection of viral RNA in only one out of six tumors in our study could be explained by the fact that the subcutaneous BxPC3 tumors are less perfused than those in the liver or breast cancer model, but also by the immune status of the mice. In contrast to these reported studies in immune competent mice, the immune deficient mice in our study did not induce an antiviral antibody response. Several studies have shown that the presence of anti-viral antibodies may increase the efficacy of oncolytic viro-immunotherapy (Adair et al., 2012; Ricca et al., 2018; Roulstone et al., 2015). Altogether, our study demonstrates that IV injections with NDV F3aa results in anti-tumor efficacy in an immune deficient subcutaneous murine model, within 10 days of treatment, and suggest this administration route is applicable to treat metastasized tumors. However, additional studies are needed to show the effect of IV injections with NDV F3aa on tumor regression at later time points in both immune deficient and immune competent murine models for PDAC.

The absence of any tissue pathologies or weight loss upon IT or IV injections with NDV F3aa in this model is in agreement with results from other studies using oncolytic NDV in immune deficient and immune competent murine models (Ch’ng et al., 2015; Jiang et al., 2018; Li et al., 2011; Liu et al., 2020; Matuszewska et al., 2019; Silberhumer et al., 2010; Wei et al., 2015; Zamarin et al., 2018). In addition, these data agree with data obtained from our study in cynomolgus macaques. This study in immune competent cynomolgus macaques demonstrated that IV injections with wild type NDV or NDV F3aa did not cause abnormalities in the animals (Buijs et al., 2014a). All together, these data indicate that IV injections with NDV F3aa does not cause health implications in these mammalian hosts.

However, systemic spread of the virus upon IV injections with NDV F3aa could potentially result in shedding of virus by the patients, which poses a potential threat to wild birds or the poultry industry (Dortmans et al., 2011). Here, we did not observe viral shedding in the saliva, urine or feces upon IV and IT injections of immune deficient tumor bearing mice. However, small amounts of viral RNA were observed in the lungs and kidneys of IV injected mice, and it cannot be ruled out that the amount of viral RNA that was shed was below the detection limit of the qRT-PCR assay. The detection limit of the assay was previously determined at 10^2^-10^4^ copies (Wise et al., 2004). In our assays the limit of detection was determined to be 0.5 TCID_50._ Previous studies have shown that low amounts of viral RNA can be detected in clinical specimens collected in virus transport medium from cynomolgus macaques, mice and chickens, including fecal samples and cloacal, nose and throat swabs. In clinical trials, where patients suffering from incurable solid tumors were IV injected with wildtype NDV, viral shedding in saliva was observed (Laurie et al., 2006). Upon IV injection of cynomolgus macaques with NDV F3aa viral shedding was also observed, however there was no effective viral transmission to naive animals and these animals did not demonstrate any sign of viral infection (Buijs et al., 2014a). These data indicate that IV injections with NDV in a mammalian host leads to minimal amounts of shedding. Whether this limited amount of shedding poses a threat to the environment needs further evaluation in clinical studies.

In conclusion, both IT and IV injections with NDV F3aa resulted in anti-tumor effects in a xenograft athymic murine model for PDAC during 10 days after the first injection. The efficacy needs confirmation in an immune competent model for PDAC, as well as at later time points. However, this study shows that both IT and IV treatment with NDV F3aa were safe in this immune deficient mammalian model, indicating that oncolytic viro-immunotherapy using NDV F3aa as oncolytic virus can be safely used to treat PDAC patients.

## Declarations

### Author contribution statement

J. Fréderique de Graaf: Conceived and designed the experiments; Performed the experiments; Analyzed and interpreted the data; Wrote the paper.

Marco Huberts: Performed the experiments; Wrote the paper.

Daphne Groeneveld & Stefan van Nieuwkoop: Performed the experiments; Analyzed and interpreted the data.

Casper H.J. van Eijck: Conceived and designed the experiments; Contributed reagents, materials, analysis tools or data.

Ron A.M. Fouchier & Bernadette G. van den Hoogen: Conceived and designed the experiments; Analyzed and interpreted the data; Wrote the paper.

### Funding statement

Dr Bernadette G ​van den Hoogen, Prof ​Ron A.M. Fouchier and Stefan van Nieuwkoop were supported by NWO-TTW [15414].

J. Fréderique de Graaf was supported by Overleven met Alvleesklier kanker [SOAK 16.01].

### Data availability statement

Data included in article/supp. material/referenced in article.

### Declaration of interest’s statement

The authors declare no conflict of interest.

### Additional information

Supplementary content related to this article has been published online at https://doi.org/10.1016/j.heliyon.2022.e09915.
